# Rotaviruses in Wild Ungulates from Germany, 2019–2022

**DOI:** 10.3390/microorganisms11030566

**Published:** 2023-02-24

**Authors:** Nadine Althof, Eva Trojnar, Reimar Johne

**Affiliations:** German Federal Institute for Risk Assessment, Max-Dohrn-Straße 8-10, 10589 Berlin, Germany

**Keywords:** rotavirus A, wild boar, roe deer, fallow deer, detection rate, genotyping

## Abstract

Rotavirus A (RVA) is an important cause of diarrhea in humans and animals. However, RVA in wild animals has only scarcely been investigated so far. Here, the presence of RVA in wild ungulates hunted between 2019 and 2022 in Brandenburg, Germany, was investigated using real-time RT-PCR and sequencing of RT-PCR products. By analyzing intestinal contents, RVA-RNA was detected in 1.0% (2/197) of wild boar (*Sus scrofa*), 1.3% (2/152) of roe deer (*Capreolus capreolus*), and 2.1% (2/95) of fallow deer (*Dama dama*) but not in 28 red deer (*Cervus elaphus*) samples. Genotyping identified G3P[13] strains in wild boar, which were closely related to previously described pig and wild boar strains. Genotype G10P[15] strains, closely related to strains from roe deer, sheep, or cattle, were found in roe deer. The strains of fallow deer represented genotype G3P[3], clustering in a group containing different strains from several hosts. The results indicated a low prevalence of RVA in wild ungulates in Germany. Associations of specific genotypes with certain ungulate species seem to exist but should be confirmed by analyses of more samples in the future.

## 1. Introduction

Rotaviruses are an important cause of infectious gastroenteritis, mainly affecting children and young animals. For humans, rotavirus infections caused approximately 128,500 children’s deaths in 2016 worldwide [1]. In domestic animals, such as pigs, cattle, horses, cats, dogs, chickens, and turkeys, rotaviruses are widely distributed, causing acute diarrhea, and chronic runting and stunting syndromes [2,3]. Recently, rotaviruses have also been described in wild animals, including bats, rodents, shrews, wild boars, and red foxes [4,5,6,7,8,9,10].

Rotaviruses belong to the family *Sedoreoviridae* and have a genome of 11 segments of double-stranded RNA [11]. Each segment encodes either one of the structural proteins VP1 to VP4, VP6, and VP7 or the non-structural proteins NSP1 to NSP5 [12]. The outer capsid proteins VP4 and VP7 are the most important targets for neutralizing antibodies and humoral immunity [13]. Based on antibody reactivity and sequence identity of VP6, the rotavirus species A to D and F to J, as well as the putative species K and L, have been identified so far [14,15]. However, rotavirus A (RVA) is considered to be the most important species regarding enteric diseases in humans and animals [12]. For RVA, a genotyping system targeting all 11 genome segments has been established, which reflects the high genetic diversity of this virus [16]. For example, for the outer capsid protein-encoding segments, 42 G-types (for Glycolylated protein VP7) and 58 P-types (for Proteolytically cleaved protein VP4) have been defined so far [17].

Specific G/P combinations of RVA genotypes are typically associated with distinct host species. However, zoonotic transmissions of rotaviruses between different hosts have also been described frequently [18,19]. In addition, the exchange of genome segments between animal and human RVA strains by reassortment events is common, resulting in new combinations of the rotavirus genome segments with potentially new virus properties [18,19].

In contrast to humans and domestic animals, RVA in wild animals has only scarcely been investigated so far. In fact, only a few data has been published on the distribution of RVA in wild ungulates, including species frequently hunted and serving as food. So far, the presence of RVA has been described in wild boars from Japan, the Czech Republic, and Croatia [9,20,21], in roe deer from Slovenia [22,23], in water deer from South Korea [24], as well as in two unspecified deer samples [25,26].

The aim of this study was, therefore, to further investigate the presence of RVA infections in wild ungulates. Fecal samples from wild boar and three deer species from Germany were analyzed by real-time RT-PCR, and the detected RVAs were characterized by the determination of their G- and P-types as well as by sequence comparisons with other RVA strains. The results indicate a circulation of RVA in German wild ungulates with low prevalence and suggest an association of specific genotype combinations with distinct host species.

## 2. Materials and Methods

### 2.1. Samples

**Figure 1:**
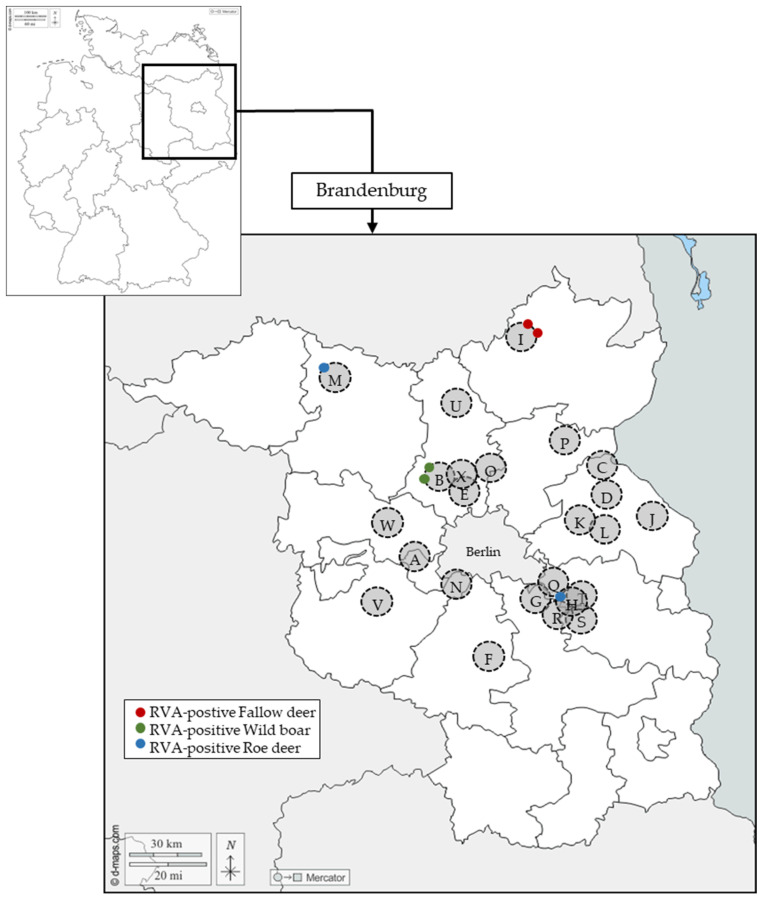
Location of the sampling sites of wild ungulates within different hunting areas (A–X) in Brandenburg, Germany. RVA-RNA-positive samples are shown by colored dots, as indicated in the box. The map was generated using d-maps.com (https://d-maps.com/carte.php?num_car=17879 and https://d-maps.com/carte.php?num_car=6198&lang=en accessed on 14 December 2022).

In total, 197 samples from wild boar (*Sus scrofa*), 152 samples from roe deer (*Capreolus capreolus*), 95 samples from fallow deer (*Dama dama*), and 28 samples from red deer (*Cervus elaphus*) were obtained during winter seasons between 2019 and 2022 from regular hunts in 24 areas in Brandenburg, Germany. More details on the distribution of samples according to the animal species and hunting regions are presented in Supplementary Table S1. In addition, a map illustrating the location of the sampling sites is presented in Figure 1. In the 2019/2020 hunting season, animals of all ages were investigated. However, as RVA-positive samples were exclusively found in young animals, only animals with an age of <3 years were sampled in the following hunting seasons. The age of the animals was estimated by weight estimation and teeth inspection performed by a veterinarian. Intestinal content was obtained from the rectum directly after hunting, and samples were stored at −20 °C until further analysis.

### 2.2. Nucleic Acid Extraction and Real-Time RT-PCR

Intestinal contents were diluted 1:10 with phosphate-buffered saline and roughly vortexed for 2 min. After centrifugation at 4000× *g* for 2 min at room temperature, RNA was extracted from 100 µL of the supernatant using the NucliSense platform on an E-MAG device (Biomerieux, Marcy-l’Étoile, France). Subsequently, RVA-specific real-time RT-PCR was performed with primers and a probe as described by Pang et al. [27] using the QuantiTect probe RT-PCR Kit (Qiagen, Hilden, Germany). In order to monitor and ensure successful RNA extraction, as well as real-time RT-PCR performance, a defined amount of Mengovirus vMC0 [28] was added to every diluted fecal sample, thereby serving as an external process control.

### 2.3. Genotyping of RVA Strains

Samples positive for RVA by real-time RT-PCR were subjected to a cascade of RT-PCRs for the generation of longer products for sequencing and subsequent genotyping. For the determination of G-Type, the RT-PCR products and the nested PCR products according to the EuroRotaNet protocol [29] were separately analyzed on ethidium bromide-stained agarose gels. The ~880 bp long product of the RT-PCR was preferentially analyzed, but if no product was visible, the ~300 bp product of the nested PCR was used. If no product was visible using this approach, the nested RT-PCR, according to Mijatovich-Rustempasic et al. [30], was attempted, which amplifies a ~200 bp product. For the determination of P-type, the RT-PCR, according to Theuns et al. [31], amplifying an ~800 bp product, was used. If no product was visible, the nested RT-PCR product of ~210 bp length, amplified according to Mijatovich-Rustempasic et al. [30], was used. The products were purified using the Monarch DNA Gel Extraction Kit (New England Biolabs GmbH, Frankfurt, Germany) and subjected to Sanger sequencing by a commercial supplier (Eurofins Genomics Germany GmbH, Ebersberg, Germany). The generated nucleotide sequences have been submitted to the GenBank database with accession numbers OQ161693–OQ161703. Genotypes were determined from the sequences by the Rotavirus A Genotyping Tool Version 0.1 (https://www.rivm.nl/mpf/typingtool/rotavirusa/ accessed on 14 December 2022) and by Nucleotide BLAST search (https://blast.ncbi.nlm.nih.gov/Blast.cgi accessed on 14 December 2022) of the closest relatives in the NCBI GenBank database.

### 2.4. Calculation of Nucleotide Sequence Identities and Phylogenetic Analysis of RVA Sequences

Nucleotide sequence identities were calculated after alignment with the Clustal W method using the MegAlign Pro 17 module of the DNASTAR software package (Lasergene, Madison, WI, USA). Phylogenetic trees were generated by the Maximum Likelihood method (parameters: 1000 bootstrap replications, Tamura-Nei model as the optimal nucleotide substitution model, uniform rates among sites, all sites used) using MEGA X version 10.1.7 [32]. The corresponding genotype reference strains [17] and the six closest relatives determined by Nucleotide BLAST search (https://blast.ncbi.nlm.nih.gov/Blast.cgi, accessed on 14 December 2022) were included in the analysis, as well as human strain Wa, turkey strain Ty-3 and common shrew strain KS11-0893 as outgroup strains. Trees were manually labeled and formatted using Microsoft Powerpoint.

## 3. Results

### 3.1. Detection of RVA in Samples of Wild Ungulates from Germany

A total of 472 intestinal content samples of wild ungulates hunted between 2019 and 2021 in 24 different areas of Brandenburg, Germany, were analyzed by real-time RT-PCR for the presence of RVA-RNA. Out of these, six samples (1.3%) tested positive. Figure 1 shows the location of all sampling areas and where positive samples were identified. According to animal species, 2 of 197 (1.0%) wild boar samples, 2 of 152 (1.3%) roe deer samples, and 2 out of 95 (2.1%) fallow deer samples were tested positive, whereas all of the 28 samples from red deer were tested negative. Details on the positive samples are shown in Table 1. Most of the positive samples originated from animals with an age of <1 year. The two positive wild boar samples were obtained from the same hunting area during one hunting season. The two positive fallow deer samples originated from the same hunting area (different from those of wild boars) but from different hunting seasons. The two positive roe deer samples were collected in different hunting areas with approximately 130 km distance between them and in different hunting seasons. All positive samples showed Ct-values between 35.1 and 37.3 in real-time RT-PCR, indicating low amounts of RVA-RNA.

### 3.2. Genotyping of Detected RVA Strains

In order to amplify longer fragments for sequencing and genotyping purpose, different RT-PCR protocols were applied to the RVA-RNA-positive samples. Fragments with a length of ≥800 bp were obtained for the two wild boar samples, one fallow deer sample, and one roe deer sample (only for the P-type in this case). No RT-PCR product could be generated for the P-type of the other fallow deer sample, whereas in all other samples, fragments between 200 bp and 300 bp could be amplified. An overview of the amplicons is presented in Table 2.

Sequencing of the amplicons followed by typing using the Rotavirus A Genotyping Tool Version 0.1 identified genotype G3P[13] for the wild boar samples, G10P[15] for the roe deer samples, and G3P[3] (and G3P[x]) for the fallow deer samples.

### 3.3. Nucleotide Sequence Identities with Closely Related RVA Strains

The sequences were used to determine nucleotide sequence identities between the identified RVA strains. By comparing the VP4- and VP7-sequences of the two wild boar strains, 100% identity was evident. The strains from roe deer showed 99.7% identity for the VP4 sequence and 99.8% identity for the VP7 sequence. The fallow deer VP7 sequences were 100% identical. A comparison of the VP7 sequences between the wild boar strains and the fallow deer strains, which are all of genotype G3, showed only 80% nucleotide sequence identity.

A Nucleotide BLAST search of the GenBank database (Table 3) identified porcine strains from UK and Slovakia as closest relatives of the wild boar strains, with 92–98% identity. The roe deer sequences had the highest sequence identities of 97–99% to ovine strains from Northern Ireland and roe deer strains from Slovenia. The closest relatives of the fallow deer sequences were from a cat, a horse, and an environmental sample from Japan, India, and Slovenia, respectively, with identities between 90% and 98%.

### 3.4. Phylogenetic Analysis of Nucleotide Sequences

Phylogenetic trees were constructed for the VP7 and VP4 sequences together with closely related strains identified by the Nucleotide BLAST search of the GenBank database and genotype reference strains. Roe deer sample 537 was not included in the analysis because only very short sequences were available. Generally, the sequences of the wild ungulates from our study clustered together with the reference strains of their determined genotypes, thus confirming the typing results.

**Figure 2:**
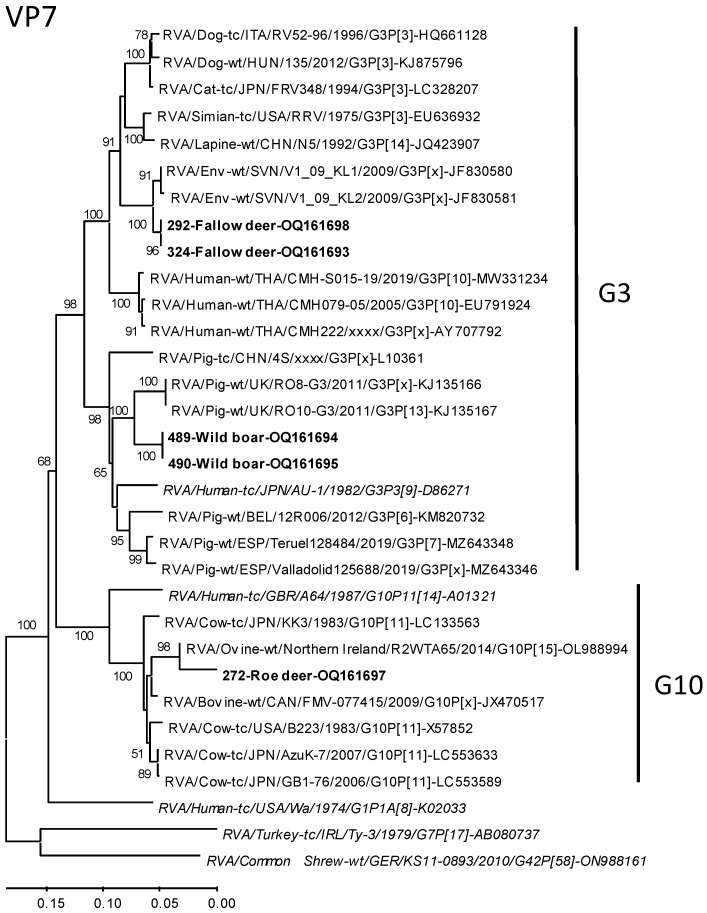
Phylogenetic relationship of the RVA strains from wild ungulates of Germany with closely related strains and genotype reference strains based on a 307 bp fragment of the VP7-encoding genome segment. The tree was constructed by the Maximum Likelihood Method using MEGA-X. Bootstrap values >50% are indicated. Scale bars indicate nucleotide substitutions per site. The strain designations and GenBank accession numbers are indicated at the branches of the trees. Strains from this study are marked in boldface, and genotype reference strains in italics. G-types are also indicated right of the tree.

In detail, the tree, based on a 307 bp fragment of the VP7 gene (Figure 2), shows a clustering of the wild boar strains together with several other pig strains as well as the human genotype reference strain. The fallow deer strains branch very closely together with environmental sequences from Slovenia, which are embedded between a cluster of human strains and a cluster of dog, cat, simian, and lapine strains. The roe deer strain clusters together with different ovine and bovine strains.

**Figure 3:**
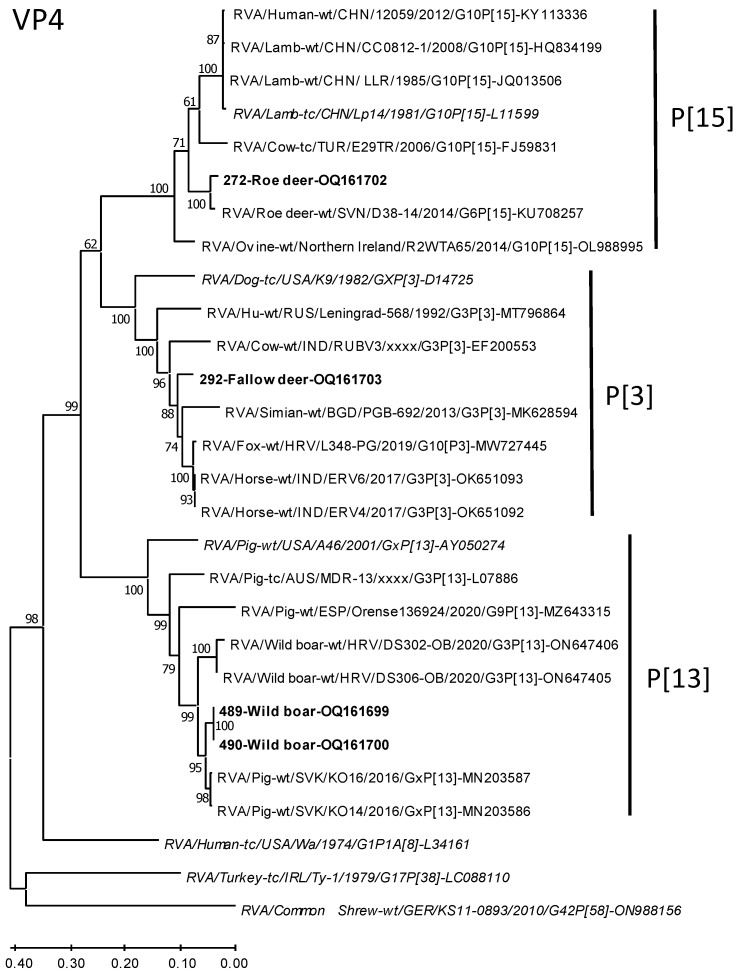
Phylogenetic relationship of the RVA strains from wild ungulates of Germany with closely related strains and genotype reference strains based on a 727 bp fragment of the VP4-encoding genome segment. The tree was constructed by the Maximum Likelihood Method using MEGA-X. Bootstrap values >50% are indicated. Scale bars indicate nucleotide substitutions per site. The strain designations and GenBank accession numbers are indicated at the branches of the trees. Strains from this study are marked in boldface, and genotype reference strains in italics. P-types are also indicated right of the tree.

In the phylogenetic tree based on a 727 bp fragment of the VP4 gene (Figure 3), the wild boar strains cluster together with strains from wild boar and pig. The fallow deer strain clusters in a diverse group of strains from cow, monkey, human, fox, horse, and dog. The roe deer strain branches together with another strain from roe deer, which both are embedded in a group of strains from ovine, cow, and human strains.

## 4. Discussion

RVA has been identified in various wild animal species, including bats, rodents, shrews, carnivores, and ungulates [4,5,6,7,8,9,10,22]. In the latter group, RVA infections in wild boar, roe deer, and water deer have been described so far [9,22,24]. In our study, we confirm the presence of RVA in wild boar and roe deer, indicating a continued circulation of this virus in these animal species. In addition, we detected RVA in fallow deer, which—to the best of our knowledge—represents the first description in this ungulate species. Further analysis of a broader range of wild animal species is necessary in the future to identify additional candidate reservoir animals, which may serve as continuous sources for RVA infection.

Generally, the detection rate in wild ungulates was low in our study. RVA was detected only in 1.0% of the investigated wild boar samples. The reported detection rates for RVA in wild boars in different countries worldwide range between 0% and 9.3% [20,21,33]. A study from the Czech Republic, which is next to Germany, detected RVA in 2.5 % of wild boars [9], which is comparable to our results. In roe deer from Slovenia, RVA was detected in 1.0 % of the samples [22], which is similar to our detection rate of 1.3%. In water deer from South Korea, an RVA detection rate of 2% was described [24], and we detected RVA in 2.1% of analyzed fallow deer samples. Generally, these detection rates are markedly lower than those described for domestic ungulates. For example, RVA detection rates in domestic pigs are reported to vary between 3.3% to 67.3% worldwide [34] or between 9.4% and 81.1% in the USA [35]. A large meta-analysis in China calculated a pooled RVA prevalence of 46% in domestic cattle [36]. These marked differences in detection rates may indicate different epidemiological settings and RVA transmission dynamics in domestic vs. wild ungulates—an assumption that requires more detailed studies, though.

An analysis of the relationship between RVA-positivity and certain hunting areas or animal species is difficult based on the data because of the low numbers of RVA-positive samples. In addition, the sampling was done using animals occurring during regular hunting and not by using a stratified sampling plan. As an example, RVA-positive fallow deer were only detected in area I, which might imply a geographically restricted occurrence of this rotavirus. However, fallow deer were only sampled in 5 of the 24 hunting areas (Supplementary Table S1), and a comparatively high number of these samples (28/95) originated from area I. The data basis is better for wild boars, where 197 samples were retrieved from 20 areas, and only two RVA-positive samples originated from area B in which 18 wild boars were analyzed. Although this finding suggests that the presence of RVA in wild boars is not evenly distributed among the hunting areas, more samples have to be analyzed in a more systematic way to prove this assumption.

The typing of the RVA strains indicated unique genotypes for each animal species. Moreover, the identified strains were closely related to already described strains from the same or similar host species, e.g., the wild boar strains to that of wild boar or pig, and the roe deer strain to that of roe deer, sheep, or cow. In roe deer, the same genotype was detected in two different hunting areas and for two different years, thus ruling out a simple area- or time-specific strain circulation. The findings may indicate an adaptation of the strains to the specific ungulate species. However, only low sample numbers have been analyzed in our study, and further studies are necessary to confirm this finding.

Genotype G3P[13] was detected in wild boars in our study. This genotype was also frequently detected in wild boars from Croatia [21], but other genotypes, e.g., G5P[13], G9P[23], or G4P[6], have also been described [9,20,21]. In roe deer, we identified genotype G10P[15], whereas G8P[14] and G6P[15] were found in other studies in this animal species [22,23]. These findings indicate that multiple RVA strains can infect wild boar and roe deer, underlying their potential to serve as reservoirs and sources of a broad range of diverse strains.

For the fallow deer strain detected in our study, clustering in a group containing diverse strains from cow, monkey, human, fox, horse, dog, and cat, as well as lapine and environmental strains, were evident. Genotype G3 has been previously detected in a wide range of animal species [5,18]. However, as no other sequences from fallow deer are available so far, it cannot be conclusively clarified if these animals were infected with a strain originating from another animal species or if it represents a so far unknown fallow deer-specific strain.

Despite the indication of host adaptation, the interspecies transmission of RVA strains—also to humans—has been repeatedly described [18,19]. Indeed, in our phylogenetic trees, several human strains also cluster near the wild ungulate strains, which may indicate possible zoonotic transmission. Generally, wild animals are known to serve as reservoir animals for several zoonotic pathogens [37]. Therefore, further screening of wild ungulates for RVA might enable a better risk assessment for RVA transmission to domestic animals or humans.

## 5. Conclusions

The study showed that RVA is present in wild ungulates in the Brandenburg region of Germany. Wild ungulates can therefore serve as a source of infection for other animals and possibly also for humans. However, in line with other studies, the detection rates were low, indicating low RVA transmission kinetics in wild ungulates. Specific genotype combinations were detected in particular animal species, which might indicate some host adaptation of the strains. However, the close relationship of the strains to other human and animal strains indicates a zoonotic potential of the detected strains. To assess the virus distribution in individual animal species and elucidate their epidemiological role, further screening of wild ungulates for RVA is necessary.

## Figures and Tables

**Table 1:** Details of RVA-positive samples from wild ungulates from Germany.

Sample Number	Animal Species	Age Group	Hunting Area	Year of Sampling	RVA-Specific Ct Value
324	Fallow deer	2–3 years	I	2019	35.9
489	Wild boar	1–2 years	B	2019	35.1
490	Wild boar	<1 year	B	2019	35.5
537	Roe deer	<1 year	M	2019	35.4
272	Roe deer	<1 year	H	2021	35.8
292	Fallow deer	<1 year	I	2021	37.3

**Table 2:** Results of the RT-PCRs and nested PCRs for genotyping of RVAs from wild ungulates from Germany. The determined genotypes are indicated in brackets.

Sample Number	Animal Species	RT-PCR [29] ~880 bp (G-Type)	Nested PCR [29] ~300 bp (G-Type)	Nested PCR [30] ~200 bp (G-Type)	RT-PCR [31] ~800 bp (P-Type)	Nested PCR [30] ~210 bp (P-Type)
324	Fallow deer	-	+ (G3)	+ (G3)	-	-
489	Wild boar	+ (G3)	+ (nd ^1^)	+ (nd)	+ (P[13])	+ (nd)
490	Wild boar	+ (G3)	+ (nd)	+ (nd)	+ (P[13])	+ (nd)
537	Roe deer	-	-	+ (G10)	-	+ (P[15])
272	Roe deer	-	+ (G10)	+ (nd)	+ (P[15])	+ (nd)
292	Fallow deer	+ (G3)	+ (nd)	+ (nd)	+ (P[3])	+ (nd)

^1^ nd: not determined.

**Table 3:** Closest relatives of RVA strains of wild ungulates from Germany according to Nucleotide BLAST search of the GenBank database.

Sample Number	Animal Species	VP7 Gene		VP4 Gene	
		Identity (%)	Strain (GenBank Acc.-No.)	Identity (%)	Strain (GenBank Acc.-No.)
324	Fallow deer	98	RVA/Env-wt/SVN/ V1_09_KL1/2009/G3P[x] (JF830580)	-	-
489	Wild boar	92	RVA/Pig-wt/UK/ RO8-G3/2011/G3P[x] (KJ135166)	98	RVA/Pig-wt/SVK/ KO16/2016/GxP[13] (MN203587)
490	Wild boar	92	RVA/Pig-wt/UK/ RO8-G3/2011/G3P[x] (KJ135166)	98	RVA/Pig-wt/SVK/ KO16/2016/GxP[13] (MN203587)
537	Roe deer	97	RVA/Ovine-wt/Northern Ireland/R2WTA65/2014/G10P[15] (OL988994)	99	RVA/Roe deer-wt/SVN/ D38-14/2014/G6P[15] (KU708257)
272	Roe deer	97	RVA/Ovine-wt/Northern Ireand/R2WTA65/2014/G10P[15] (OL988994)	98	RVA/Roe deer-wt/SVN/ D38-14/2014/G6P[15] (KU708257)
292	Fallow deer	90	RVA/Cat-tc/JPN/ FRV348/1994/G3P[3] (LC328207)	94	RVA/Horse-wt/IND/ ERV6/2017/G3P[3] (OK651093)

## Data Availability

Nucleotide sequence data are available at the GenBank database with the accession numbers as presented in Section 2.3. of the manuscript. Additional data can be retrieved upon request from R.J.

## Supplementary Materials

The following supporting information can be downloaded at: https://www.mdpi.com/article/10.3390/microorganisms11030566/s1, Supplementary Table S1: Sample numbers according to the hunting area and animal species.
