# Supplementary material for: Rotaviruses in Wild Ungulates from Germany, 2019–2022
Source: Microorganisms. 2023 Feb 24;11(3):566. doi: 10.3390/microorganisms11030566 (PMC10058221; doi:10.3390/microorganisms11030566)
Supplement: Supplementary file 1 [file microorganisms-11-00566-s001.zip › microorganisms-2239744-supplementary.pdf]

Supplementary Table S1 for Article:

Rotaviruses in wild ungulates from Germany, 2019-2022

Nadine Althof , Eva Trojnar and Reimar Johne

Supplementary Table S1: Sample numbers according to the hunting area and animal species.

Hunting area	Name of Hunting area (administrative district)	Fallow deer samples	Wild boar samples	Roe deer samples	Red deer samples
**A**	**Kartzow** (Potsdam-Mittelmark)	0	16	16	0
**B**	**Rüthnicker Heide** (Oberhavel)	57	18	1	1
**C**	**Hohensaaten/Hohenfinow** (Märkisch-Oderland)	0	1	8	0
**D**	**Biesow-Harnekop** (Märkisch-Oderland)	0	13	16	6
**E**	**Lehnitz/Borgsdorf** (Oberhavel)	0	27	8	10
**F**	**Horstwalde** (Teltow-Fläming)	4	14	13	0
**G**	**Niederlehme West/Krummeluch** (Dahme-Spreewald/Oder-Spree)	0	15	7	0
**H**	**Markgrafpieske** (Oder-Spree)	0	3	6	0
**I**	**Zerweliner Heide** (Uckermark)	28	9	5	1
**J**	**Platkow** (Märkisch-Oderland)	0	1	5	0
**K**	**Wilkendorf** (Märkisch-Oderland)	1	9	7	2
**L**	**Klosterdorf** (Märkisch-Oderland)	0	3	0	0
**M**	**Wittstock/Rossower Heide** (Ostprignitz-Ruppin)	0	22	9	6
**N**	**Güterfelde** (Potsdam-Mittelmark)	0	6	8	0
**O**	**Klosterfelde** (Oberhavel)	0	0	4	0
**P**	**Chorin** (Barnim)	0	0	4	0
**Q**	**Niederlehme Ost/Rauen** (Oder-Spree)	0	1	2	0
**R**	**Streganz** (Dahme-Spreewald)	0	9	11	1
**S**	**Storkow/Grubenmühle** (Oder-Spree)	0	13	14	1
**T**	**Neu-Stahnsdorf** (Oder-Spree)	0	1	2	0
**U**	**Barsdorf/Tornow** (Oberhavel)	0	0	2	0
**V**	**Lehnin** (Potsdam-Mittelmark)	5	3	1	0
**W**	**Groß-Behnitz** (Havelland)	0	13	2	0
**X**	**Oranienburg** (Oberhavel)	0	0	1	0
	**Total**	**95**	**197**	**152**	**28**
